# Comparative Mitochondrial Genome Analysis of the Intestinal Schistosomiasis Snail Host *Biomphalaria pfeifferi* from Multiple Populations in Gezira State, Sudan

**DOI:** 10.3390/ijms26104756

**Published:** 2025-05-16

**Authors:** Arwa Osman, Peter S. Andrus, Xianglu Zhu, Zhaoyang Dong, Yunhai Guo, Bakri Y. M. Nour, Xiaonong Zhou, Liming Zhao

**Affiliations:** 1 State Key Laboratory of Bioreactor Engineering, East China University of Science and Technology, Shanghai 200237, China; y10180460@mail.ecust.edu.cn (A.O.);; 2 Blue Nile National Institute for Communicable Diseases, University of Gezira, P.O. Box 20, Wad Madani 5118 40466, Sudan; 3 National Institute of Parasitic Diseases, Chinese Center for Disease Control and Prevention (Chinese Center for Tropical Diseases Research), Shanghai 200025, China; 4 School of Global Health, Chinese Centre for Tropical Diseases Research, Shanghai Jiao Tong University School of Medicine, Shanghai 200025, China; 5 Faculty of Medical Laboratories, University of Gezira, P.O. Box 20, Wad Madani 5118 40466, Sudan

**Keywords:** mitogenome, *Biomphalaria pfeifferi*, comparative analysis, phylogenetic relationship, Gezira State, Sudan

## Abstract

*Biomphalaria pfeifferi* is a key intermediate host for *Schistosoma mansoni* transmission in Sudan. In total, 27 complete mitochondrial genomes from seven *B. pfeifferi* populations in Gezira State, Sudan, were sequenced for the first time to investigate their population structure and phylogenetic relationships. This involved comparing the nucleotide composition, codon usage, rRNAs, and tRNAs of the East Gezira (EG), South Gezira (SG), Hasahisa (HA), Greater Wad Medani (GW), Managil (MA), and North Umelgura (NU1, NU3) populations. All 27 mitogenomes (13,688–13,696 bp) contained 37 genes with conserved AT/GC content (76.7/23.4%). Phylogenetic analysis revealed that although samples clustered within the same clade, *B. pfeifferi* from EG, SG, NU1, and NU3 grouped closely with *B. pfeifferi* from Kenya, whereas HA and GW samples formed distinct ancestral lineages. The MA population exhibited unique genetic characteristics, supported by phylogenetic trees and nucleotide/amino acid identity, suggesting the potential presence of a distinct *B. pfeifferi* subspecies that warrants further investigation. All protein-coding genes evolved under negative selection, with the amino acids of *nad1* and *nad6* being highly conserved, while *nad3* exhibited some variation. Further research on the mitogenomic diversity of *B. pfeifferi* and other *Biomphalaria* species in Sudan and across Africa is needed in order to better understand the population structure and evolutionary history of *Biomphalaria*.

## 1. Introduction

*Biomphalaria* (Gastropoda: Planorbidae) is a genus of freshwater snails responsible for the transmission of the parasitic blood fluke *Schistosoma mansoni*, the leading cause of intestinal schistosomiasis in humans [1,2,3]. Although the majority of African *Biomphalaria* species have been found naturally infected in the field, only 18 of the identified *Biomphalaria* species are susceptible to *S. mansoni* infection [1,4,5,6]. Phylogenetic studies of the African species found that only *B. camerunensis* and *B. pfeifferi* were well defined, while the six other *Biomphalaria* species (*B. alexandrina*, *B. angulosa*, *B. choanomphala*, *B. smithi*, *B. stanleyi*, and *B. sudanica*) formed a poorly defined group called the ‘Nilotic species complex’ [6,7]. In Sudan, *B. pfeifferi* and *B. sudanica* are the most important intermediate hosts of intestinal schistosomiasis. *Biomphalaria pfeifferi* has been reported in the past in different areas in Sudan, including the Blue Nile State, the White Nile State, North Sudan, and Gezira State [8,9,10,11,12]. At present, it is found in Gezira State [13,14,15], Kassala State [16,17], Gedarif State [18], Sennar State [19], White Nile State [20], and the River Nile State [21]. Intestinal schistosomiasis remains a major public health concern in Gezira State, and crucial integrated control measures are needed. A nationwide survey conducted in 2017 by the Federal Ministry of Health (FMOH), supported by the Korea International Cooperation Agency (KOICA) across the 18 States of Sudan, including Gezira State, where 106 schools and 6307 students were examined, reported *S. mansoni* prevalence as 2.6% [22]. In 2023, the prevalence increased to 8.6% in Gezira State in the follow-up survey by the FMOH and KOICA [personal communication]. The transmission of the disease is heavily dependent on the intermediate snail host’s (*Biomphalaria* spp.) presence in the Gezira State irrigation systems, as this provides an ideal breeding site for snails and disease transmission [5,12,23].

The mitochondrial genomes (mitogenomes) of gastropods exhibit similar structures, being composed of circular double-stranded DNA molecules that contain 13 protein-coding genes (PCGs), two ribosomal RNAs (rRNA), 22 transfer RNAs (tRNA), a potential control region (CR) [24], and a large number of overlapping gene boundaries [25]. The mitochondrial genome is a valuable tool for understanding evolution and phylogenetic relationships due to the preservation of its structure and composition [26]. This is due to mitochondrial genes having only maternal inheritance, a high mutation rate, and a low recombination rate, which make them an effective genetic marker to study species identification and phylogenetic relationships [27,28,29,30]. Several factors may contribute to the genetic structure of *Biomphalaria* populations, including self-fertilization, environmental conditions, host–parasite interactions, and parasitic infections [1,6,31,32,33,34]. Furthermore, periodic droughts and flooding in irrigation canals could have resulted in population bottlenecks, influencing genetic structure and diversity [2,31,35,36,37]. Various studies have examined the population structure and phylogeny of several *Biomphalaria* species utilizing mitochondrial gene fragments, such as the Cytochrome Oxidase Subunit I (COI) gene and the 16S ribosomal RNA (16S rRNA) component, as well as popular nuclear markers like the internal transcribed spacer (ITS1 and ITS2) sequences [2,7,31,38,39,40,41,42,43,44,45,46]. However, the utilization of the complete mitochondrial genome offers more insights than individual gene fragments in phylogenetic reconstruction studies [47]. The first mitogenome of the major schistosome-transmitting snail genera to be sequenced was *Biomphalaria glabrata* in 2004 (NC_005439; [48]), followed by *Oncomelania hupensis* in 2010 (NC_013187; [49]), and *Bulinus truncatus* in 2021 (MT947902; [50]). Subsequently, the annotated mitogenomes of several *Biomphalaria* species have been sequenced, such as *B. glabrata*, *B. pfeifferi*, and *B. choanomphala* [51], *B. sudanica* [51,52], *B. tenagophila* [53] and *B. straminea* [54]. Moreover, studies on the complete annotated genomes of *B. glabrata* [55,56,57], *B. straminea* [58] and *B. pfeifferi* [32] have also been published, which offer more insight into the phylogenetic relationships and evolutionary history of the genus.

Studying the genetic composition and population structure of *Biomphalaria* snails in Sudan is important, as the genetic diversity and constitution of a population can affect their susceptibility to schistosome infection, influence disease transmission rates, and can help the design of more effective control strategies. Therefore, the aim of this study was to sequence the whole mitochondrial genomes of multiple *B. pfeifferi* snails obtained from Gezira State, Sudan, to examine their population structure, and compare it to other *Biomphalaria* species.

## 2. Results

### 2.1. Mitochondrial DNA Characteristics, Structure and Organization

**Figure 1:**
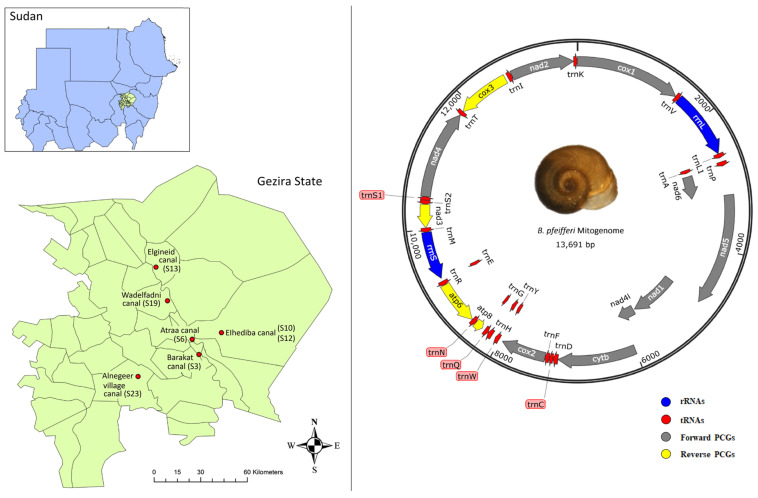
Map of Sudan and the *Biomphalaria pfeifferi* collection sites of Gezira State (**left**; sites: Barakat canal S3 [SG], Atraa canal S6 [GW], Elhediba canal S10 [NU1] and S12 [NU3], Elgineid canal S13 [EG], Wadelfadni canal S19 [HA], and Alnegeer village canal S23 [MA]). This map was created using the Geographic Information System (GIS) software ArcGIS10.5 (Esri, CA, USA; https://www.arcgis.com/ accessed on 3 December 2024). An example circular mitochondrial genome map of *B. pfeifferi* from Gezira State, Sudan (**right**; site 13 EG) is shown as an example. Protein coding genes (PCGs), ribosomal (rRNAS), and tRNA (tRNAs) genes are shown with standard abbreviations [nad: NADH dehydrogenase subunit; cytb: cytochrome b; cox: cytochrome c oxidase subunit. atp: atp synthase F0 subunit: 12S: small subunit ribosomal RNA; 16S: large subunit ribosomal RNA]. Genes oriented in the clockwise direction indicate forward gene transcription (+), while genes oriented in the counterclockwise direction indicate reverse gene transcription (−). This map was created using SnapGene v8.0.2 software (www.snapgene.com).

The mitochondrial assembly results showed that all of the 27 *B. pfeifferi* sequences obtained by NOVOPlasty were circular (Figure 1). The total sequence length of the mitogenomes ranged from 13,688 to 13,696 bp, with an average length of 13,692 bp. The genomic GC content was between 23.3% and 23.4%, with an average value of 23.35% (Table 1). The mitochondrial annotation results showed that the twenty-seven samples consisted of thirteen Protein Coding Genes (PCGs), twenty-two tRNA genes and two rRNA genes, giving a total of 37 genes. Nine protein-coding genes (*nad5*, *nad1*, *nad4l*, *cytb*, *cox2*, *nad4*, *nad2*, *cox1*, and *nad6*), the *rrnL* (*16S* rRNA) gene, and fourteen tRNA genes were located on the plus (heavy) strand, while four protein-coding genes (*atp6*, *atp8*, *nad3* and *cox3*), the *rrnS* (*12S* rRNA) gene, and eight tRNA genes were located on the minus (light) strand. Of the 27 mitochondrial genomes, one sample from each of the seven areas was selected for further analysis due to high similarity among samples (Table 1). The total base pair composition of the entire mitogenome for the seven samples was almost identical, with A = 33.7–33.8%, T = 42.8–43%, C = 10.2–10.4%, and G = 13–13.1%. They had a high AT content of 76.6–76.7%, a GC content of 23.3–23.4%, a negative AT skew of ≈−0.12, and a positive GC skew of ≈0.11–0.12. The overall nucleotide composition was biased toward AT. The highest AT content (81.7–81.9%) was observed in *nad2* and the highest GC content was observed in *cox1* (29.1–30%). While the highest GC skew (0.36–0.37) was observed in the *nad6* gene. The A + T content of the total rRNAs was higher than that of the total tRNAs and total PCGs. The AT skew was negative in all genes, and all PCGs showed positive GC skew values, except for *atp6* and *atp8*, which showed negative values. However, the MA population showed a positive value in *atp8* (0.032). In addition, only the MA sample showed a negative GC skew value in the *nad3* gene (−0.031). The G + C content had equal values (9.3%) in sites EG, SG, NU1, and NU3, resulting in a GC skew of zero. Overall, the nucleotide compositions of the EG, SG, NU1, and NU3 samples were similar to each other. Likewise, the HA and GW samples were more similar to each other. Conversely, the MA sample showed different compositions in the *atp6*, *atp8*, *nad1*, *nad2*, *nad3*, and *nad4* genes compared to the other populations (Table 1; Supplementary File S1; Table S1).

### 2.2. Overlapping and Non-Coding Regions

Non-coding regions were observed across the mitogenome and ranged from 1 to 41 bp in length. However, the number of intragenic regions varied between areas, ranging from 8 bp (NU1, SG), 9 bp (GW, HA, MA), 10 bp (NU3), and 11 bp (EG). The longest intergenic region in all samples (41 bp) was located between the *cox3* and *trnI* (gau). This may serve as a transcriptional regulatory area, as it is characterized by high AT content and repetitive T stretches and forms a hairpin (stem-loop) structure, revealing conserved motifs resembling mitochondrial promoter elements. The number of overlapping regions was eight in samples EG, GW, HA, NU3, and MA, and nine in samples NU1 and SG. They ranged from 1 to 53 bp in all samples, with the longest overlap (53 bp) being the region of *cytb* overlapping *nad4l* (Supplementary File S2: Dataset S1).

### 2.3. Protein-Coding Genes (PCGs)

The total length of the 13 PCGs was 10,695 bp in all populations. The length of the 13 PCGs ranged from 123 bp (*atp8*) to 1656 bp (*nad5*). When comparing the start codons for each population, we found that all of the PCGs started with either ATT, ATG, or ATA codons, except for the *cox1*, which started with TTG. For the stop codons, six of the PCGs were terminated with TAA, six had incomplete stop codons (T), and one gene used TAG (Table 2, Supplementary File S2: Dataset S1). The standard invertebrate mitochondrial genetic code was used for all start and stop codons.

### 2.4. Transfer RNA and Ribosomal RNA Analysis

**Figure 2:**
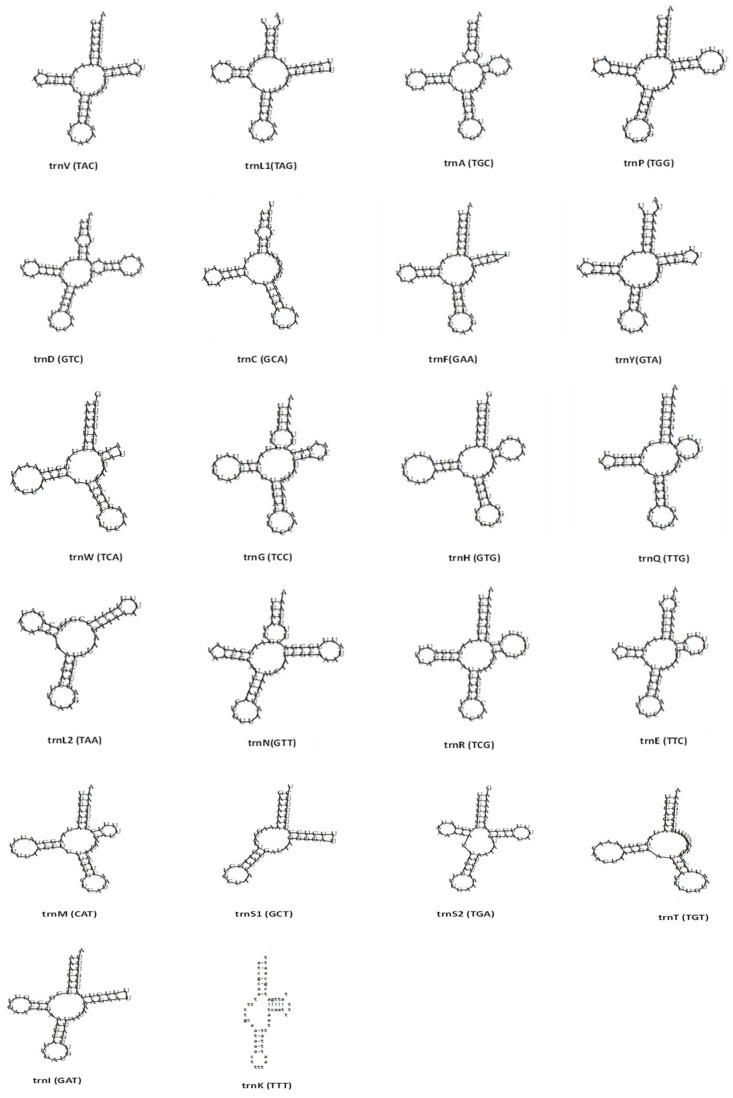
Clover leaf secondary structures of the 22 tRNAs from the Sudanese Gezira State *B. pfeifferi* mitogenomes. Structure prediction was done using MITOS2, tRNAscan-SE v2.0, and ARWEN v1.2.

Nineteen out of the 22 tRNAs were successfully annotated using software, while three tRNAs (*trnG*, *trnS2*, and *trnK*) could not be detected automatically. This is likely due to the atypical and highly divergent structures of these tRNAs in gastropod mitogenomes compared to other animals. As a result, these three tRNAs were identified manually through nucleotide alignment using their counterparts in *B. pfeifferi* and *B. sudanica* mitogenomes [51]. The length of the 22 transfer RNA genes slightly varied between samples, ranging from 49 bp (*trnK*) to 70 bp (*trnD*). The overall AT content of the tRNAs ranged from 77.3% to 77.5%, showing an AT bias. When comparing the tRNA genes between our seven samples, we found that each corresponding amino acid is encoded by the same anticodon, except *trnS1* (serine 1), which uses the anticodon GCT (Table 2). The majority of the *B. pfeifferi* tRNAs displayed a typical clover leaf secondary structure with four arms (acceptor, D, TΨC, and anticodon arms). However, structural anomalies were observed in several of the tRNAs across all populations. Specifically, the D-arm was absent in *trnL2*, *trnK*, and *trnS1*, while the TΨC-arm was missing in *trnC* and *trnT*. Additionally, a reduced TΨC-arm was observed in *trnL1*, *trnS1*, *trnF*, *trnW*, and *trnI* (Figure 2). These consistent structural deviations suggest potential modifications in tRNA processing or function in our Sudanese *B. pfeifferi* populations.

**Figure 3:**
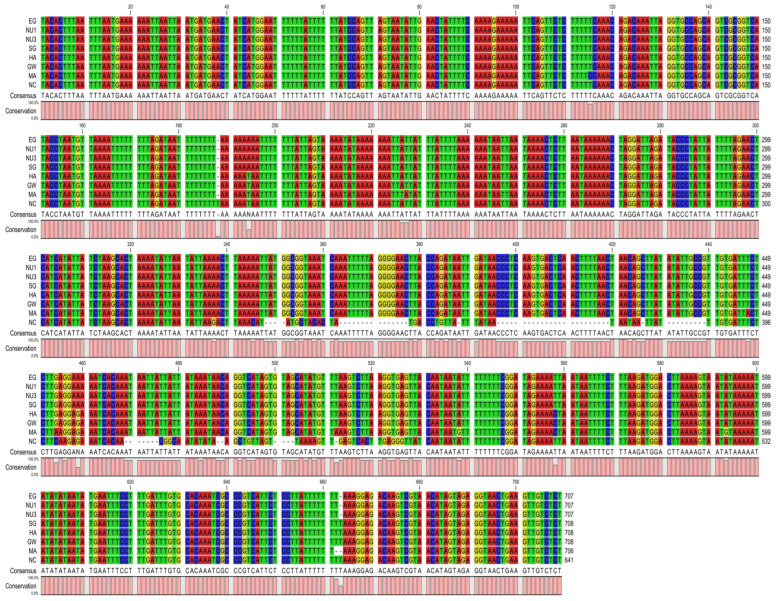
12S rRNA sequence alignment of the Gezira State and Kenyan *B. pfeifferi* (NC_038059) samples. This alignment was performed using CLC Genomics workbench v25.0.1 (https://digitalinsights.qiagen.com/).

**Figure 4:**
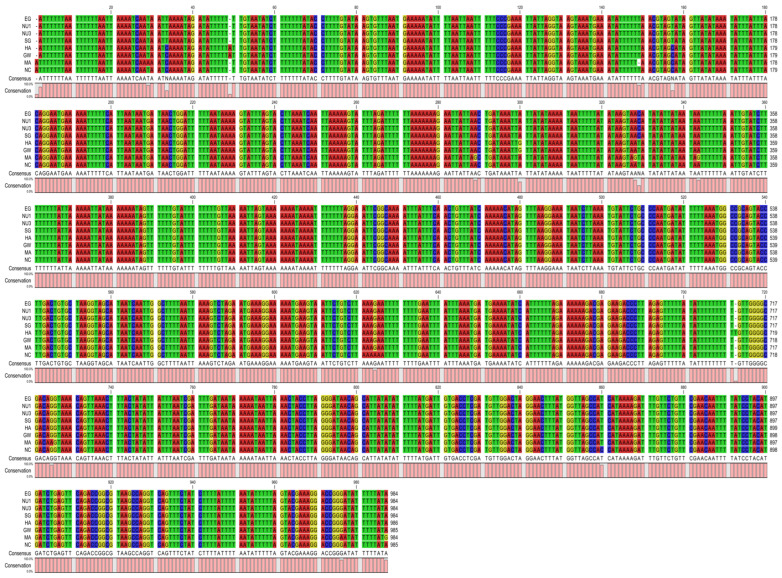
16S rRNA sequence alignment of the Gezira State and Kenyan *B. pfeifferi* (NC_038059 *) samples. This alignment was performed using CLC Genomics workbench v25.0.1 (https://digitalinsights.qiagen.com/). Note: * The length of the 16S rRNA was re-annotated and corrected, as the original annotation overlapped the trnV and trnA genes.

The 12S ribosomal RNA had a length ranging from 706 to 708 bp and was located between *trnE* and *trnM*. The 16S ribosomal RNA had a length ranging from 984 to 986 bp and was located between *trnV* and *trnL1* (Table 2). The rRNAs had a nucleotide composition biased towards A and T with an AT percentage between 77.5 and 78%, and a GC percentage between 22.1 and 22.6% (Table 1). Their base compositions showed AT skew values of −0.019 to −0.027, and GC skew values of 0.095 to 0.098, indicating a bias towards T and G (Table 1). A pairwise comparison was calculated for the 12S and 16S rRNA genes to estimate the evolutionary divergence between *B. pfeifferi* populations. We found that 12S showed high divergence (0.063–0.072) when comparing our populations to the Kenyan *B. pfeifferi* (NC_038059), with the Kenyan *B. pfeifferi* sequence having 71 missing base pairs (Figure 3, Table 3). In contrast, divergence within the Gezira samples was low. For the 16S rRNA alignment and pairwise comparison data (Figure 4, Table 4), we found low divergence within the Gezira samples and in comparison to the Kenyan *B. pfeifferi* sequence (0.004–0.009). The higher similarity observed in the 16S rRNA likely reflects stronger functional constraints and negative selection maintaining its sequence conservation. Whereas the greater divergence in the 12S rRNA suggests a relatively relaxed selective pressure, allowing more variability such as structural deletions, particularly in comparisons with the Kenyan *B. pfeifferi* sequence.

### 2.5. Amino Acid Usage and the Relative Synonymous Codon Usage (RSCU) Analysis

**Figure 5:**
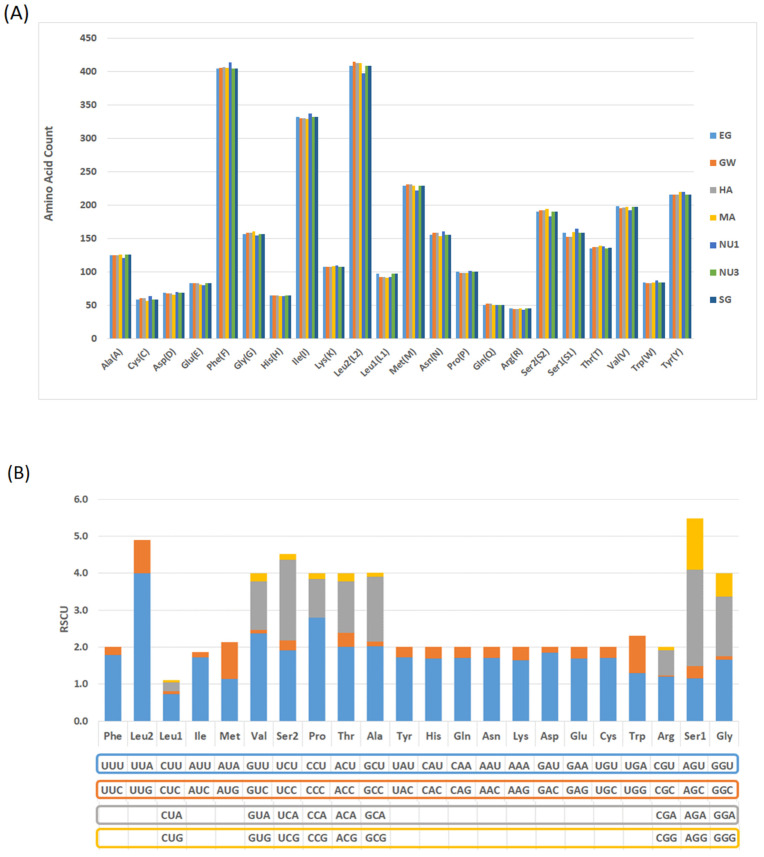
(**A**) Amino acid count in the Gezira *B. pfeifferi* mitogenomes from different areas and (**B**) the relative synonymous codon usage (RSCU). Bar graph colors indicated the makeup of specific codon sequences for each amino acid.

The amino acid usage of *B. pfeifferi* from the seven sites indicated that Leu2, Phe, and Ile were the most frequently used amino acids, with minor count variation across populations. The least used were Arg, Gln, and Cys (as shown in Figure 5A; Supplementary File S3: Figure S1). The most frequently used codons were UUA-Leu2, CCU-Pro, and GUU-Val; they all ended with A or U and each had a RSCU value over 2 across all populations (Figure 5B). Nine amino acids (Leu1, Val, Ser2, Pro, Thr, Ala, Arg, Ser1, and Gly) were encoded by four codons, and thirteen were encoded by two codons (Phe, Leu2, Ile, Met, Tyr, His, Gln, Asn, Lys, Asp, Glu, Cys, and Trp; Figure 5B).

### 2.6. Non-Synonymous and Synonymous Substitutions (Ka/Ks Ratio) Analysis

**Figure 6:**
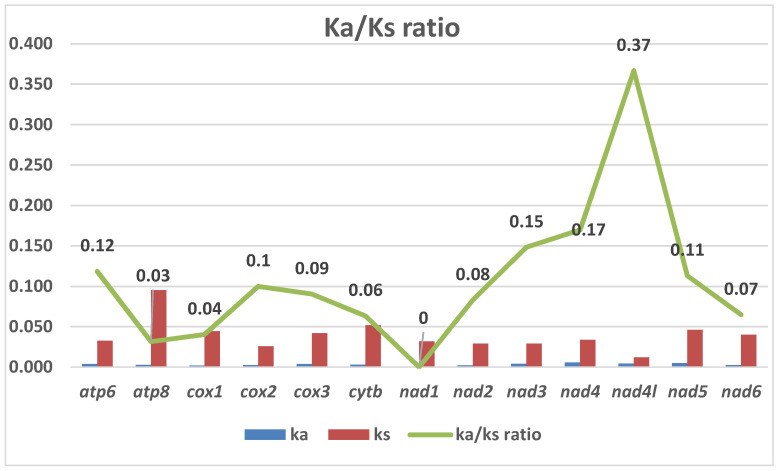
The Ka/Ks ratios for the 13 PCGs of the Gezira State *B. pfeifferi* mitogenomes.

The Ka/Ks ratio measures the rate of non-synonymous (amino acid-changing) substitutions (Ka) relative to synonymous (silent) substitutions (Ks) in protein-coding genes; ratios below 1 reflect negative (purifying) selection, where harmful mutations are eliminated to maintain protein structure and function, whereas ratios above 1 would indicate positive (diversifying) selection. Therefore, to assess the evolutionary dynamics and selection pressure in the *B. pfeifferi* mitogenomes, we calculated the Ka/Ks ratios for the 13 protein-coding genes (PCGs) from the seven Gezira State populations (Figure 6). Among these genes, the *nad4l* gene exhibited the highest Ka/Ks ratio (0.37), while the *nad1* gene had the lowest (0.0). All Ka/Ks ratios were below 1; this indicates that all of the PCGs are evolving under negative selection, suggesting that deleterious mutations affecting protein function are being selectively removed.

### 2.7. Nucleotide and Amino Acid Identity Analysis

**Figure 7:**
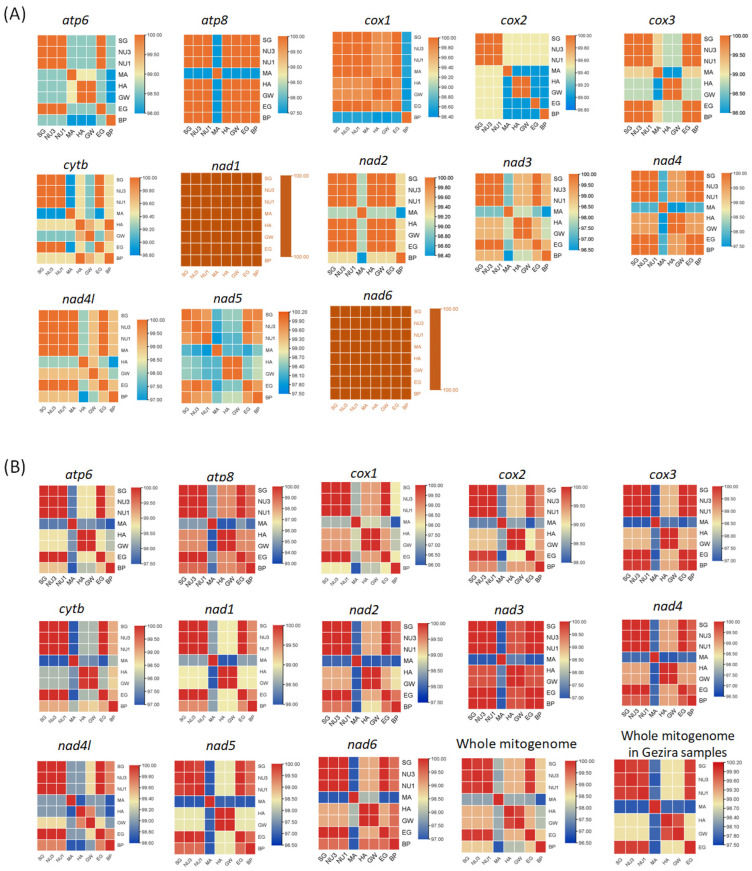
Heatmaps of the *B. pfeifferi* mitogenomes of the Gezira State and Kenya (NC_038059 *) samples showing the pairwise comparisons of (**A**) amino acid identity (%) across the 13 protein-coding genes (PCGs); and (**B**) nucleotide identity (%) across the 13 PCGs and the whole mitogenome. Pairwise heatmaps were created using TBtools-II v2.152. Note: * This analysis used a modified version of the Kenyan *B. pfeifferi* (NC_038059) reference genome, as the *cox2*, *cox3*, *nad2*, *nad3*, *nad4*, *nad6*, *atp8*, *and atp6* genes contained overlapping regions with tRNA genes. The amino acid and nucleotide sequences were re-annotated following the standard annotation procedures used to annotate our Gezira samples.

The heatmap analysis of pairwise comparisons in amino acid (AA) and nucleotide (NT) sequences (Figure 7) revealed distinct patterns of conservation and variability across the Gezira State populations. Highly conserved proteins were observed in the *nad1* and *nad6* genes (100%), despite nucleotide sequence variations among different areas. In contrast, the *nad3* gene displayed some amino acid variability (96.5–100%) in the MA, HA, and GW populations compared to other populations. The MA population was the only group showing notable amino acid diversity in the *atp8* gene (97.5%) (Figure 7A). At the nucleotide level, *nad4l* had the highest identity (98.7–100%), whereas *atp8* had the lowest (93.5–100%; Figure 7B). Overall, populations from SG, EG, NU3, and NU1 exhibited high nucleotide identity (99.9%), whereas GW, HA, and MA showed slightly lower conservation (Figure 7B; Table 1). When compared to the *B. pfeifferi* reference genome (NC_038059), the whole mitogenome sequence identity was 98.7% for sites EG, SG, NU3, and NU1; 98.1% for sites HA and GW; and 96.9% for site MA, indicating greater divergence in the Managil population.

### 2.8. Phylogenetic Analysis

**Figure 8:**
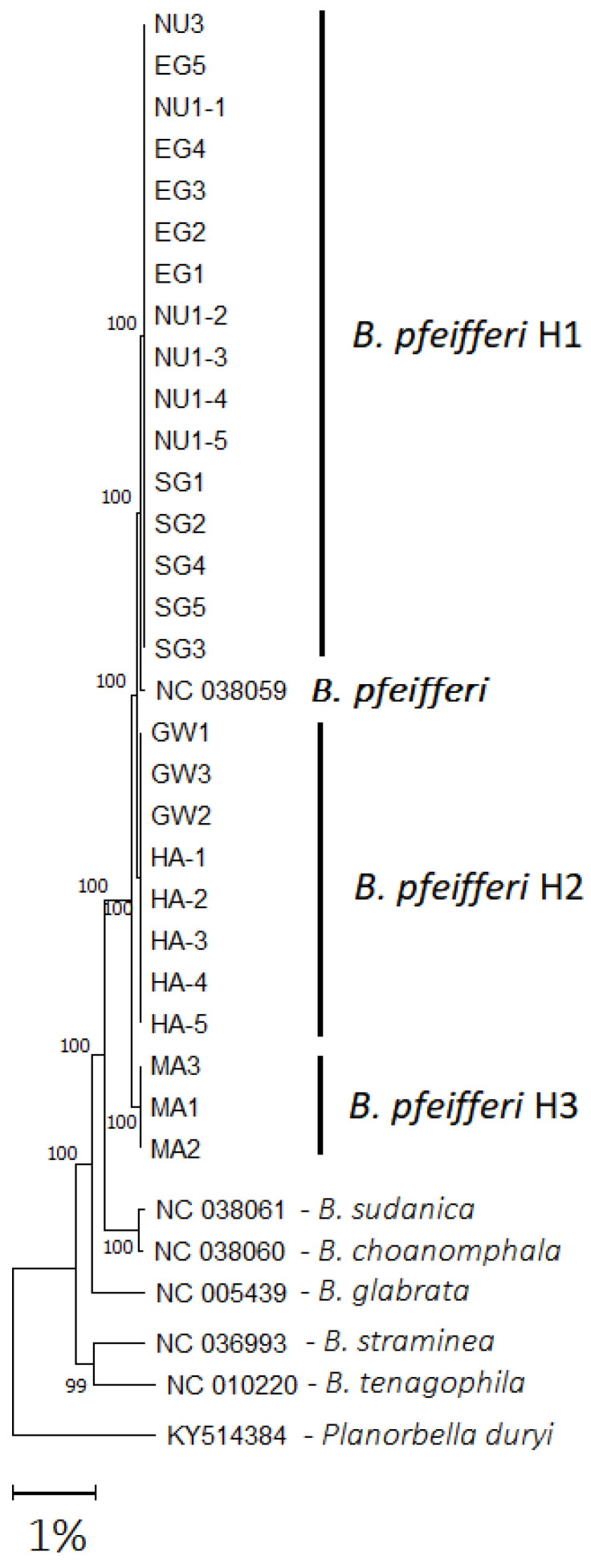
Maximum-likelihood tree of the 27 *B. pfeifferi* samples from Gezira State, and the mitochondrial genomes (10,061 bp) of available African and South American *Biomphalaria* species. This tree was generated using MEGA.11 using a GTR+Γ model and is rooted on *Planorbella duryi*. Numbers on branches indicate the bootstrap percentages for 1000 replicates. The scale bar represents 1% sequence divergence.

The new mitogenome sequences obtained in this study were deposited to GenBank under accession numbers PV213442–PV213448 and had total lengths ranging from 13,688 to 13,696 bp (Table 5). A maximum likelihood tree was generated using all 27 of our *B. pfeifferi* samples and six of the *Biomphalaria* spp. mitogenomes retrieved from NCBI, with *Planorbella duryi* as the out-group (Table 5, Figure 8). The 27 *B. pfeifferi* samples from Gezira made up three unique mtDNA haplotypes (H1, H2, and H3) and formed a clade with the Kenyan *B. pfeifferi* (NC_038059). Notably, sub-branches containing EG, NU1, SG, and NU3 (H1) were closest to the Kenyan *B. pfeifferi* sample. Conversely, GW and HA (H2) clustered together, suggesting a shared evolutionary history, while the MA samples (H3) showed the most unique genetic characteristics (Figure 8). Lastly, the topology and bootstrap values remained consistent for both the coding nucleotide sequence (NT), and the translated amino acid (AA) trees (Supplementary File S3: Figure S2).

## 3. Discussion

Differences in mitochondrial genome size, gene order, gene content, intergenic regions, repetitive sequences, and RNA secondary structures are key indicators of evolutionary relationships among species [30,60,61,62]. These mitogenomic variations often arise due to adaptive responses to environmental pressures [63]. Genomic research on the four major intermediate snail host genera (e.g., *Bulinus*, *Biomphalaria*, *Oncomelania*, and *Neotricula*) involved in human schistosomiasis transmission has progressed at different rates, largely driven by their importance in schistosome epidemiology and the availability of research funding. Notably, this study represents the first mitochondrial population genetic analysis of an African *Biomphalaria* species, specifically *B. pfeifferi*, one of the most important vectors of *S. mansoni* in sub-Saharan Africa. Previous mitogenomic studies on *Bulinus* and *Biomphalaria* have primarily explored interspecific differences within these genera [51], whereas our findings reveal intraspecific mitochondrial genetic variation across multiple *B. pfeifferi* populations within a defined geographical region.

The *B. pfeifferi* from Gezira State maintain the typical gene order and exhibit no major rearrangements or expansions. This conservation mirrors patterns observed previously [48,51,53,54] and suggests strong evolutionary constraints on mitochondrial architecture within the genus. Similarly, we found strong negative selection across all 13 PCGs (Ka/Ks < 1), consistent with previous *B. pfeifferi* study [51], indicating that selective pressure on mitochondrial genes is conserved within this species. Interestingly, a negative GC skew in the *nad3* gene was detected specifically in the Managil (MA) population. While negative GC skew patterns have been linked to environmental adaptation in other molluscs [64,65], this is the first report of such a shift in *B. pfeifferi*, suggesting that irrigation-driven habitat changes in Gezira may influence mutational dynamics. Moreover, structural analysis of tRNAs revealed that most *B. pfeifferi* tRNAs retain the canonical clover leaf structure, although loss and/or reductions in the D-arm or TΨC-arm were detected. These structural reductions are consistent with patterns across molluscan and metazoan mitogenomes [25,66,67,68,69,70,71], likely representing adaptations towards genome compaction while maintaining essential function.

When examining the start codon of the *cox1* gene, we found all our populations exhibited a (TTG) start codon, aligning with the South American species (e.g., *B. glabrata* strain 1742 [48], *B. tenagophila* [53], and *B. straminea* [54]). However, it differs from the ATC start codon observed in Kenyan *B. pfeifferi* and the ATG codon in *B. sudanica* and *B. choanomphala* [51]. Our study confirms the frequent occurrence of incomplete stop codons (T) in six genes (*atp6*, *cox2*, *cox3*, *nad2*, *nad3,* and *nad4*), a phenomenon commonly observed in invertebrate mitogenomes and present in other *Biomphalaria* species [48,53,54]. These incomplete stop codons are linked to the compact nature of mitochondrial DNA, which has evolved to minimize genome size by omitting full stop codons in some protein-coding genes [72]. Instead, during post-transcriptional processing, these truncated codons are completed by polyadenylation, where a poly-A tail is added to the mRNA, turning the incomplete uracil (U) into a complete stop codon (UAA). However, incomplete stop codons frequently create annotation challenges, as commonly used tools such as MitoZ and MITOS2 often misidentify gene boundaries leading to inaccurate mitogenome annotations. For example, in our analysis, we observed that several of the Zhang et al. [51] *Biomphalaria* reference genomes exhibited misannotations, in which eight PCGs (*cox2*, *cox3*, *nad2*, *nad3*, *nad4*, *nad6*, *atp8*, and *atp6*) and the 16S *rrnL* overlapped with adjacent tRNA genes, requiring corrections before use.

Phylogenetic comparisons showed high similarity between the Gezira State and the Kenyan (NC_038059) *B. pfeifferi* sequences, supporting previous findings of limited divergence across African populations [51,73]. However, subtle differences in gene length, nucleotide composition, and amino acid sequences were observed. We found high divergence in the 12S rRNA when comparing the Gezira samples with the Kenyan sequence. This divergence is likely due to the Kenyan strain having been maintained under laboratory conditions for several years, whereas the Gezira populations remain wild and exposed to natural selective pressures, including habitat modification and parasitic infections. The slightly greater divergence observed in the Managil (MA), Hasahisa (HA), and Greater Wad Medani (GW) populations suggests localized adaptation or restricted gene flow, and may result from geographical isolation and/or irrigation-driven bottlenecks [2,37]. Similar intraspecific genetic variation was also observed in *B. glabrata* [48]. The highest nucleotide sequence variation was observed in the Managil (MA) population, potentially for several reasons: (I) a genetically distinct founder population introduced by humans or birds with limited gene flow; (II) local adaptation to specific environmental pressures in irrigation systems; and/or (III) the presence of a distinct *B. pfeifferi* subspecies, warranting further investigation.

## 4. Materials and Methods

### 4.1. Sample Collection

Natural populations of *B. pfeifferi* were collected from seven sites across six localities in Gezira State, Sudan, between December 2022 and March 2023 (Table 6, Figure 1). Gezira State, located in an agricultural region, is highly endemic for both urinary and intestinal schistosomiasis [22,74]. Sites with high amounts of human and/or animal contact and *Biomphalaria*-favorable water conditions were selected. Geographical coordinates (longitude and latitude) for each site were recorded using a Global Positioning System (GPS) device. Snails were collected using the scooping method by trained public health personnel, following WHO guidelines [75,76]. Subsequently, snails were transported to the Medical Entomology and Vector Control Department Laboratory at the Blue Nile National Institute for Communicable Diseases, University of Gezira, Sudan. Specimens were sorted and identified to species level based on shell morphology, following standard identification keys [77,78]. Snails were individually preserved in labeled tubes containing 95% ethanol and stored at −20 °C. Molecular laboratory analyses were then performed at the National Institute of Parasitic Diseases, China CDC, in Shanghai, People’s Republic of China.

### 4.2. DNA Extraction

A total of 27 *B. pfeifferi* snails were selected for DNA extraction and mitochondrial genome sequencing (Table 6). Each snail was individually crushed, and the soft tissue was soaked in distilled water overnight to remove residual ethanol. Samples were then air-dried for 4 hrs prior to DNA extraction. Genomic DNA from 16 snails was extracted using the Qiagen DNeasy Blood and Tissue Kit (Lot: 175021295, Cat. No. 69506, QIAGEN GmbH, Hilden, Germany) according to the manufacturer’s protocol. The remaining 11 samples were processed using the standard CTAB (Cetyltrimethylammonium Bromide), Phenol–Chloroform–Isoamyl alcohol extraction method [51]. All DNA samples were stored at −20 °C after extraction until further use for sequencing.

### 4.3. Mitochondrial Genome Sequencing, Assembly and Annotation

We examined the main characteristics of the *B. pfeifferi* mitogenomes, including their nucleotide composition, codon utilization, rRNAs and the secondary structure of their tRNAs. Mitochondrial genome sequencing and data generation were conducted by OneMore Tech (Wuhan Wanmo Technology Co., Ltd., Wuhan, China). Genomic DNA was randomly fragmented and size-selected to an appropriate average fragment length. The fragments were then end-repaired, 3′ adenylated, and ligated with sequencing adapters. PCR amplification was subsequently performed. Single-stranded circular DNA molecules were generated via rolling circle amplification [79], producing DNA nanoballs (DNBs) each containing over 300 copies of the circularized DNA. The DNBs were loaded onto patterned nanoarrays using high-density DNA nanochip technology and sequenced via combinatorial probe-anchored synthesis (cPAS) technology [80]. Raw image data were converted into sequence reads through base calling, and the results were saved in FASTQ format, including base quality scores.

Data filtering and redundancy removal were conducted using Fastp v0.23.2 [81], and sequencing quality was assessed using FastQC v0.11.9 [82] with default settings. Mitochondrial genome assembly was performed using NOVOPlasty v4.3.1 [83] with the following parameters: genome size range = 10,899–16,348 bp, K-mer = 33, read length = 150 bp, insert size = 300 bp, and paired-end reads (PE). The resulting 27 assembled mitochondrial genomes were annotated using both MitoZ v3.6 [84] (annotate clade Mollusca) and MITOS2 (http://mitos2.bioinf.uni-leipzig.de/index.py accessed on 10 December 2024) [72] with the invertebrate mitochondrial genetic code. Annotations were manually corrected using NCBI ORFfinder (https://www.ncbi.nlm.nih.gov/orffinder/ accessed on 10 December 2024), and circular maps of the mitochondrial genomes were generated with SnapGene v8.0.2 software (www.snapgene.com).

### 4.4. Transfer RNAs and Ribosomal RNAs Analysis

The secondary structures of tRNA genes were predicted using ARWEN v1.2 (http://130.235.244.92/ARWEN/ accessed on 15 December 2024)) [85], the tRNAscan-SE v2.0 web server (https://lowelab.ucsc.edu/tRNAscan-SE/ accessed on 15 December 2024)) [86], and MITOS2 [72]. Pairwise nucleotide comparisons of rRNAs and the 13 PCGs were performed using MEGA.11 [87]. rRNA alignment was performed using CLC Genomics workbench v25.0.1 (QIAGEN, Aarhus, Denmark; https://digitalinsights.qiagen.com/).

### 4.5. Amino Acid Usage and Relative Synonymous Codon Usage RSCU Analysis

Nucleotide composition, codon usage of protein-coding genes (PCGs), and relative synonymous codon usage (RSCU) were analyzed using MEGA.11 [87] and an online RSCU analysis tool (https://jamiemcgowan.ie/bioinf/rscu.html, accessed on 24 January 2025). The AT and GC skew values were calculated using the formulas AT skew = (A − T)/(A + T) and GC skew = (G − C)/(G + C).

### 4.6. Ka/Ks Ratio, Nucleotide and Amino Acid Identity Analysis

The ratio of non-synonymous (Ka) to synonymous (Ks) substitutions for mitochondrial PCGs was estimated using DnaSP v6 [88]. Amino acid (AA) and nucleotide (NT) sequence identities for the 13 PCGs were calculated using the online Sequence Manipulation Suite (https://www.bioinformatics.org/sms2/ident_sim.html, accessed on 30 January 2025) [89]. Heatmaps for AA and NT identities were generated using TBtools-II v2.152 [90].

### 4.7. Phylogenetic Analysis

Phylogenetic analyses were conducted to assess the relationships between the 27 *B. pfeifferi* samples and six additional *Biomphalaria* spp. mitochondrial genome sequences retrieved from NCBI, with *Planorbella duryi* used as an out-group (Table 5). A maximum likelihood (ML) tree was constructed based on multiple sequence alignment of the complete mitochondrial genomes using MEGA v11. The best-fit substitution model was determined using the model test function in MEGA v11, based on the lowest Bayesian Information Criterion (BIC) score, and the GTR model was selected. The ML tree was generated with 1000 bootstrap replicates to assess the robustness of the phylogenetic relationships. Additionally, multiple sequence alignments of the coding DNA sequences (CDS) and corresponding protein sequences of the 13 mitochondrial PCGs were performed using MUSCLE v3.8.31 [91]. Two additional ML phylogenetic trees, based on the concatenated coding sequences (CDSs) and protein sequences of the 13 PCGs, were constructed using RAxML v8.2.12 [92].

The mitochondrial genome of *B. pfeifferi* (NC_038059) is currently the only publicly available complete mitogenome for this species. However, several protein-coding genes were inaccurately annotated, including instances of gene overlap with adjacent tRNA genes and overestimation of coding sequence lengths. To ensure reliable comparative analyses with our newly generated data, we manually re-annotated the reference mitogenome following standard mitochondrial annotation guidelines. Specifically, we corrected the annotations and gene boundaries of the *16S rRNA*, *atp8*, *atp6*, *nad2*, *nad3*, *nad4*, *nad6*, *cox2*, and *cox3* genes.

## 5. Conclusions

The complete mitochondrial genome sequences of 27 Sudanese *Biomphalaria pfeifferi* snails from seven populations were characterized and compared. The EG, SG, NU1, and NU3 populations exhibited high genetic similarity; in contrast, the HA and GW populations showed some divergence. Notably, the MA population displayed distinct genetic variation, as supported by phylogenetic analysis, nucleotide identity, and amino acid sequence comparisons. These findings suggest that the MA population may represent a distinct subspecies of *B. pfeifferi*, warranting further investigation. Despite the genetic similarities of the EG, SG, NU1, and NU3 populations to the Kenyan *B. pfeifferi* reference mitogenome, significant variation was observed in the 12S *rrnS* sequence between the Kenyan and Gezira samples. This highlights the need for further studies to explore the genetic diversity, evolutionary history, and phylogenetic relationships of *B. pfeifferi* and other *Biomphalaria* populations across Sudan and Africa.

## Figures and Tables

**Table 1:** Nucleotide composition of *B. pfeifferi* samples from EG, GW, HA, MA, NU1, NU3, and SG localities, Gezira State, Sudan.

Regions	Sample	A	C	G	T	AT (%)	GC (%)	AT Skew	GC Skew
Whole mitogenome	EG	33.8	10.4	13.0	42.8	76.6	23.4	−0.117	0.111
	GW	33.8	10.3	13.0	42.9	76.7	23.3	−0.119	0.116
	HA	33.8	10.3	13.0	42.9	76.7	23.3	−0.119	0.116
	MA	33.7	10.2	13.1	43.0	76.7	23.3	−0.121	0.124
	NU1	33.8	10.4	13.0	42.8	76.6	23.4	−0.117	0.111
	NU3	33.8	10.4	13.0	42.8	76.6	23.4	−0.117	0.111
	SG	33.8	10.4	13.0	42.8	76.6	23.4	−0.117	0.111
rRNAs	EG	38.1	10.0	12.1	39.9	78.0	22.1	−0.023	0.095
	GW	37.9	10.1	12.3	39.8	77.7	22.4	−0.024	0.098
	HA	37.8	10.1	12.3	39.9	77.7	22.4	−0.027	0.098
	MA	38.0	10.2	12.4	39.5	77.5	22.6	−0.019	0.097
	NU1	38.1	10.0	12.1	39.9	78.0	22.1	−0.023	0.095
	NU3	38.1	10.0	12.1	39.9	78.0	22.1	−0.023	0.095
	SG	38.0	10.0	12.1	39.9	77.9	22.1	−0.024	0.095
tRNAs	EG	36.9	9.8	12.9	40.4	77.3	22.7	−0.045	0.137
	GW	37.0	9.8	12.7	40.5	77.5	22.5	−0.045	0.129
	HA	36.9	9.8	12.7	40.6	77.5	22.5	−0.048	0.129
	MA	36.8	9.9	12.8	40.6	77.4	22.7	−0.049	0.128
	NU1	36.8	9.9	12.8	40.5	77.3	22.7	−0.048	0.128
	NU3	36.9	9.9	12.9	40.4	77.3	22.8	−0.045	0.132
	SG	36.7	9.9	12.8	40.6	77.3	22.7	−0.05	0.128
PCGs	EG	30.6	10.5	13.3	45.6	76.2	23.8	−0.197	0.118
	GW	30.5	10.4	13.3	45.8	76.3	23.7	−0.2	0.122
	HA	30.5	10.4	13.3	45.8	76.3	23.7	−0.2	0.122
	MA	30.6	10.4	13.3	45.8	76.4	23.7	−0.199	0.122
	NU1	30.6	10.5	13.3	45.6	76.2	23.8	−0.197	0.118
	NU3	30.6	10.5	13.3	45.6	76.2	23.8	−0.197	0.118
	SG	30.6	10.5	13.3	45.6	76.2	23.8	−0.197	0.118

**Table 2:** Annotation of the seven Gezira State *B. pfeifferi* population mitogenomes.

Gene Name	Strand	NU3 (bp)	NU1 (bp)	SG (bp)	EG (bp)	GW (bp)	HA (bp)	MA (bp)	Codon (Start/Stop)	Anticodon
*cox1*	+	1527	1527	1527	1527	1527	1527	1527	TTG/TAA	
*trnV(uac)*	+	61	61	61	61	61	61	60		TAC
*16S rrnL*	+	984	984	984	984	985	986	984		
*trnL1(uag)*	+	63	63	63	63	63	63	63		TAG
*trnA(ugc)*	+	63	63	63	63	63	63	63		TGC
*trnP(ugg)*	+	64	64	64	64	65	66	63		TGG
*nad6*	+	435	435	435	435	435	435	435	ATT/TAA	
*nad5*	+	1656	1656	1656	1656	1656	1656	1656	ATG/TAG	
*nad1*	+	891	891	891	891	891	891	891	ATG/TAA	
*nad4l*	+	306	306	306	306	306	306	306	ATA/TAA	
*cytb*	+	1128	1128	1128	1128	1128	1128	1128	ATA/TAA	
*trnD(guc)*	+	69	69	69	69	67	70	69		GTC
*trnC(gca)*	+	56	58	58	56	58	56	58		GCA
*trnF(gaa)*	+	61	61	61	61	61	61	61		GAA
*cox2*	+	652	652	652	652	652	652	652	ATA/T	
*trnY(gua)*	+	61	61	61	61	61	61	61		GTA
*trnW(uca)*	+	62	62	62	62	63	63	63		TCA
*trnG(ucc)*	+	63	63	63	63	63	63	63		TCC
*trnH(gug)*	+	68	68	68	68	66	66	64		GTG
*trnQ(uug)*	−	58	58	58	58	58	58	59		TTG
*trnL2(uaa)*	−	52	54	54	52	52	52	52		TAA
*atp8*	−	123	123	123	123	123	123	123	ATT/TAA	
*trnN(guu)*	−	66	66	66	66	67	67	67		GTT
*atp6*	−	643	643	643	643	643	643	643	ATT/T	
*trnR(ucg)*	−	64	64	65	64	64	64	65		TCG
*trnE(uuc)*	−	58	58	58	58	57	57	58		TTC
*12S rrnS*	−	707	707	708	707	708	708	706		
*trnM(cau)*	−	63	63	63	63	63	63	63		CAT
*nad3*	−	343	343	343	343	343	343	343	ATT/T	
*trnS2(uga)*	−	61	61	61	61	62	62	63		TGA
*trnS1(gcu)*	+	54	54	53	54	53	53	53		GCT
*nad4*	+	1303	1303	1303	1303	1303	1303	1303	ATA/T	
*trnT(ugu)*	−	63	63	63	63	63	63	63		TGT
*cox3*	−	775	775	775	775	775	775	775	ATA/T	
*trnI(gau)*	+	64	64	64	64	64	64	64		GAT
*nad2*	+	913	913	913	913	913	913	913	ATT/T	
*trnK(uuu)*	+	51	51	52	51	53	53	49		TTT

**Table 3:** 12S rRNA pairwise comparison and percent identity (%) matrix of the Gezira State and the Kenyan *B. pfeifferi* (NC_038059) samples.

	EG	NU1	NU3	SG	HA	GW	MA	NC_038059
EG								
NU1	0 (**100**)							
NU3	0 (**100**)	0 (**100**)						
SG	0 (**99.9**)	0 (**99.9**)	0 (**99.9**)					
HA	0.0042 (**99.4**)	0.0042 (**99.4**)	0.0042 (**99.4**)	0.0043 (**99.6**)				
GW	0.0042 (**99.4**)	0.0042 (**99.4**)	0.0042 (**99.4**)	0.0043 (**99.6**)	0 (**100**)			
MA	0.0156 (**98.3**)	0.0156 (**98.3**)	0.0156 (**98.3**)	0.0156 (**98.2**)	0.0113 (**98.6**)	0.0113 (**98.6**)		
NC_038059	0.064 (**84.3**)	0.064 (**84.3**)	0.064 (**84.3**)	0.064 (**84.5**)	0.063 (**84.6**)	0.063 (**84.6**)	0.072 (**83.5**)	

Note: Percentage identities (%) are in bold.

**Table 4:** 16S rRNA pairwise comparison and percentage identity (%) matrix of the Gezira State and Kenyan *B. pfeifferi* (NC_038059 *) samples.

	EG	NU1	NU3	SG	HA	GW	MA	NC_038059 *
EG								
NU1	0 (**100**)							
NU3	0 (**100**)	0 (**100**)	0 (**100**)					
SG	0 (**100**)	0 (**100**)	0 (**100**)					
HA	0.0041 (**99.4**)	0.0041 (**99.4**)	0.0041 (**99.4**)	0.0041 (**99.4**)				
GW	0.0041 (**99.5**)	0.0041 (**99.5**)	0.0041 (**99.5**)	0.0041 (**99.5**)	0 (**99.9**)			
MA	0.0112 (**98.7**)	0.0112 (**98.7**)	0.0112 (**98.7**)	0.0112 (**98.7**)	0.0092 (**98.7**)	0.0092 (**98.8**)		
NC_038059 *	0.006 **(99.3**)	0.006 (**99.3**)	0.006 (**99.3**)	0.006 (**99.3**)	0.004 (**99.3**)	0.0041 (**99.4**)	0.0091 (**98.9**)	

Note: Percentage identities (%) are in bold. * The length of the 16S rRNA (*rrnL*) was re-annotated and corrected, as the original annotation overlapped the *trnV* and *trnA* genes.

**Table 5:** List of *Planorbidae* mitogenomes analyzed in this study with their GenBank accession numbers.

Species Name	Accession Number	Genome Size (Base Pair)	Reference
*Biomphalaria pfeifferi* Sudan (EG area)	PV213442	13,691	This study
*B. pfeifferi* Sudan (GW area)	PV213443	13,694	
*B. pfeifferi* Sudan (HA area)	PV213444	13,696	
*B. pfeifferi* Sudan (MA area)	PV213445	13,688	
*B. pfeifferi* Sudan (NU1 area)	PV213446	13,691	
*B. pfeifferi* Sudan (NU3 area)	PV213447	13,690	
*B. pfeifferi* Sudan (SG area)	PV213448	13,693	
*B. pfeifferi* (Kenya) *	NC_038059	13,624	Zhang et al. [51]
*B. Sudanica* (Kenya) *	NC_038060	13.671	Zhang et al. [51]
*B. choanomphala* (Kenya) *	NC_038061	13,672	Zhang et al. [51]
*B. straminea* (China)	NC_036993	13,650	Zhou et al. [54]
*B. tenagophila* (Brazil) *	NC_010220	13,722	Jannotti-Passos et al. [53]
*B. glabrata* (UK) *	NC_005439	13,670	Dejong et al. [48]
*Planorbella duryi* (Outgroup)	KY_514384	14,217	Schultz et al. [59]

Note: * *Biomphalaria* species maintained in a laboratory setting.

**Table 6:** Site information, number of snails extracted (n = 27), and GPS coordinates for *B. pfeifferi* snails collected in Gezira State, Sudan.

Locality/Administrative Unit	Collection Site ID/ Water Body Name	No. of *Biomphalaria* Extracted	Latitude	Longitude
South Gezira/Barakat	S3 Barakat canal	5	14.357	33.526
Greater Wadmedani	S6 Atraa	3	14.445	33.487
North Umelgura/Elhediba	S10 Elhediba	5	14.484	33.658
	S12 Elhediba	1	14.484	33.657
East Gezira/Elgineid	S13 Elgineid	5	14.866	33.277
Hasahisa/Wadelfadni	S19 Wadelfadni	5	14.6704	33.343
Managil/Eboud	S23 Eboud/alnegeer village	3	14.230	33.173

## Data Availability

All data are provided within the manuscript itself. The mitochondrial genomic sequence data generated in this study is provided in GenBank with accession numbers PV213442–PV213448.

## Supplementary Materials

The following supporting information can be downloaded at https://www.mdpi.com/article/10.3390/ijms26104756/s1.

## Abbreviations

EG	East Gezira
SG	South Gezira
NU1	North Umelgura1
NU3	North Umelgura3
SG	South Gezira
HA	Hasahisa
GW	Greater Wadmedani
MA	Managil
COI	Cytochrome Oxidase Subunit I gene
16S rRNA	16S ribosomal RNA gene
12S rRNA	12S ribosomal RNA gene
ITS1 and ITS2	Internal transcribed spacers 1 and 2
tRNA	Transfer ribosomal RNA
rRNA	Ribosomal RNA
DNB	DNA nanoball
cPAS	Combinatorial probe-anchored Synthesis technology
ML	Maximum likelihood
CDS	Coding sequences
PCGs	Protein-coding genes
rrnL	16S ribosomal RNA
rrnS	12S ribosomal RNA
RSCU	Relative synonymous codon usage
Ka/Ks ratio	Non-synonymous and synonymous substitutions
bp	Base pair
