# Supplementary material for: Comparative Mitochondrial Genome Analysis of the Intestinal Schistosomiasis Snail Host Biomphalaria pfeifferi from Multiple Populations in Gezira State, Sudan
Source: Int J Mol Sci. 2025 May 16;26(10):4756. doi: 10.3390/ijms26104756 (PMC12112705; doi:10.3390/ijms26104756)
Supplement: Supplementary file 1 [file ijms-26-04756-s001.zip › ijms-3585360-supplementary/Supp. files/SUP. 3 (Figures S1 and S2).pdf]

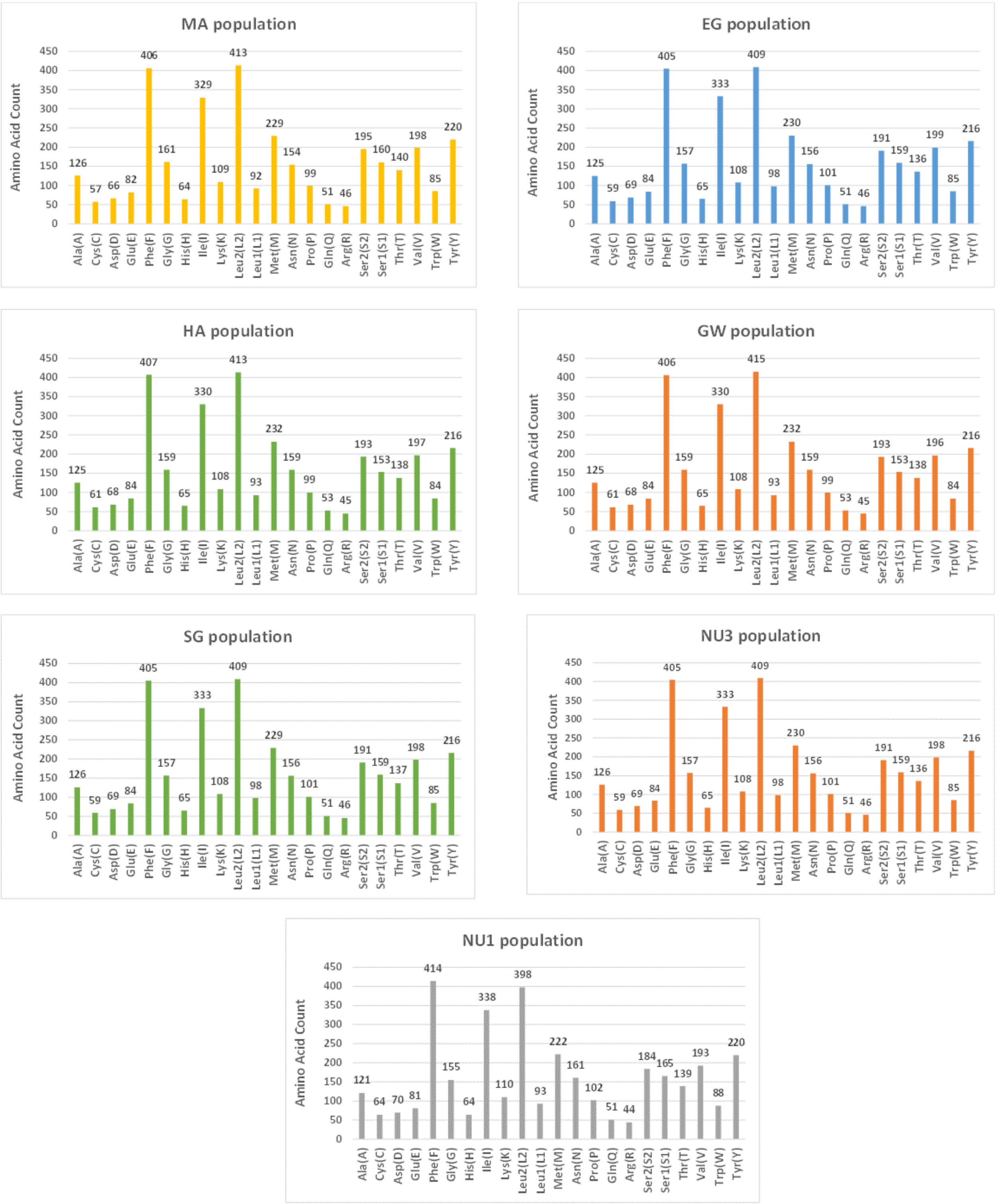

Figure S1: Amino acid count in *B.pfeifferi* mitogenome from different areas

(A) NT:

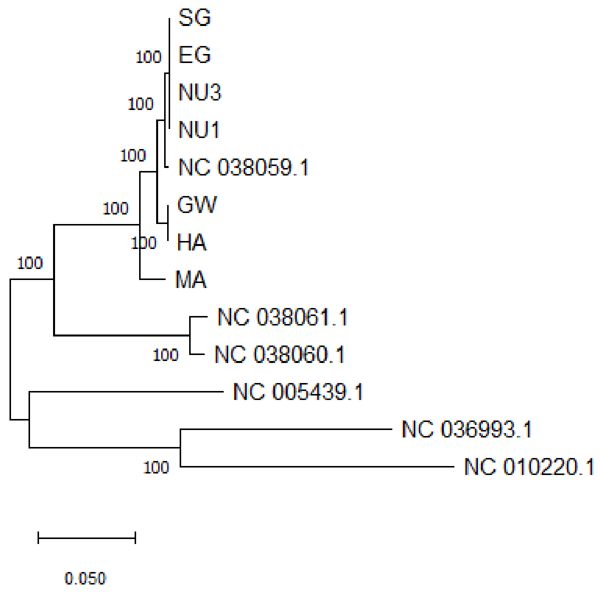

(B): AA

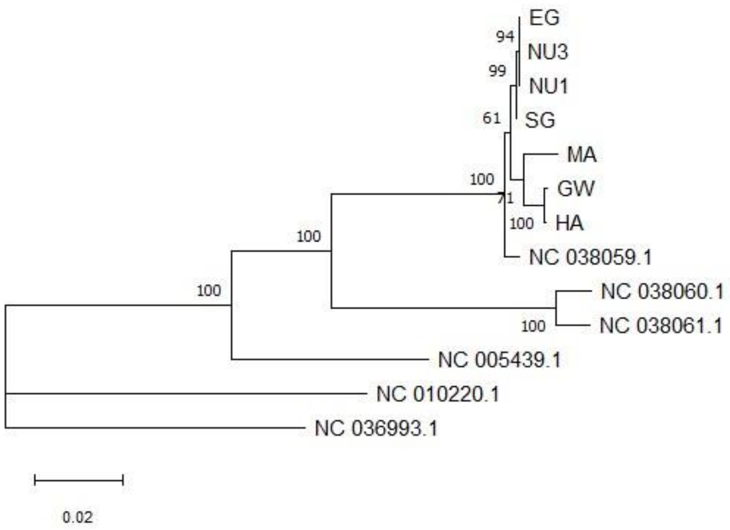

Figure S2. ML tree for the 13 PCGs (A) coding genes **and (B)** their translated amino acid
