# Supplementary material for: Comparative Mitochondrial Genome Analysis of the Intestinal Schistosomiasis Snail Host Biomphalaria pfeifferi from Multiple Populations in Gezira State, Sudan
Source: Int J Mol Sci. 2025 May 16;26(10):4756. doi: 10.3390/ijms26104756 (PMC12112705; doi:10.3390/ijms26104756)
Supplement: Supplementary file 1 [file ijms-26-04756-s001.zip › ijms-3585360-supplementary/Supp. files/SUP.1 Table S1.pdf]

Table S1. Nucleotide composition of *B. pfeifferi* samples from EG, GW, HA, MA, NU1, NU3 and SG localities, Gezira State, Sudan.

Regions	Samples	A	C	G	T	AT (%)	GC (%)	AT skew	GC skew
atp6	**EG**	32.8	11.7	10.6	44.9	77.7	22.3	-0.156	-0.049
	**GW**	32.8	11.4	10.4	45.4	78.2	21.8	-0.161	-0.046
	**HA**	32.8	11.4	10.4	45.4	78.2	21.8	-0.161	-0.046
	**MA**	33.6	12.3	10	44.2	77.8	22.3	-0.136	-0.103
	**NU1**	32.8	11.7	10.6	44.9	77.7	22.3	-0.156	-0.049
	**NU3**	32.8	11.7	10.6	44.9	77.7	22.3	-0.156	-0.049
	**SG**	32.8	11.7	10.6	44.9	77.7	22.3	-0.156	-0.049
atp8	**EG**	35.8	13.8	10.6	39.8	75.6	24.4	-0.053	-0.131
	**GW**	35	14.6	10.6	39.8	74.8	25.2	-0.064	-0.159
	**HA**	35	14.6	10.6	39.8	74.8	25.2	-0.064	-0.159
	**MA**	32.5	12.2	13	42.3	74.8	25.2	-0.131	0.032
	**NU1**	35.8	13.8	10.6	39.8	75.6	24.4	-0.053	-0.131
	**NU3**	35.8	13.8	10.6	39.8	75.6	24.4	-0.053	-0.131
	**SG**	35.8	13.8	10.6	39.8	75.6	24.4	-0.053	-0.131
cox1	**EG**	25.6	13.5	16.5	44.4	70	30	-0.269	0.1
	**GW**	25.5	13	16.5	44.9	70.4	29.5	-0.276	0.119
	**HA**	25.5	13	16.5	44.9	70.4	29.5	-0.276	0.119
	**MA**	25.7	12.7	16.4	45.2	70.9	29.1	-0.275	0.127
	**NU1**	25.6	13.5	16.5	44.4	70	30	-0.269	0.1
	**NU3**	25.6	13.5	16.5	44.4	70	30	-0.269	0.1
	**SG**	25.6	13.5	16.5	44.4	70	30	-0.269	0.1
cox2	**EG**	33.6	12.7	14.1	39.6	73.2	26.8	-0.082	0.052
	**GW**	33.6	12.7	14.1	39.6	73.2	26.8	-0.082	0.052
	**HA**	33.6	12.7	14.1	39.6	72.7	27.3	-0.082	0.052
	**MA**	33.4	12.9	14.4	39.3	72.7	27.3	-0.081	0.055
	**NU1**	33.6	12.9	14.1	39.4	73	27	-0.079	0.044
	**NU3**	33.6	12.9	14.1	39.4	73	27	-0.079	0.044
	**SG**	33.6	12.9	14.1	39.4	73	27	-0.079	0.044
cox3	**EG**	30.8	13	14.7	41.4	72.2	27.7	-0.147	0.061
	**GW**	31.2	12.9	14.3	41.5	72.7	27.2	-0.142	0.051
	**HA**	31.2	12.9	14.3	41.5	72.7	27.2	-0.142	0.051
	**MA**	31.4	12.8	14.2	41.7	73.1	27	-0.141	0.052
	**NU1**	30.8	13	14.7	41.4	72.2	27.7	-0.147	0.061
	**NU3**	30.8	13	14.7	41.4	72.2	27.7	-0.147	0.061
	**SG**	30.8	13	14.7	41.4	72.2	27.7	-0.147	0.061

cytb	**EG**	28	12.4	13.6	46	74	26	-0.243	0.046
	**GW**	27.7	12.5	14	45.8	73.5	26.5	-0.246	0.057
	**HA**	27.7	12.5	14.1	45.7	73.4	26.6	-0.245	0.06
	**MA**	28	12.1	13.7	46.3	74.3	25.8	-0.246	0.062
	**NU1**	28	12.4	13.6	46	74	26	-0.243	0.046
	**NU3**	28	12.4	13.6	46	74	26	-0.243	0.046
	**SG**	28	12.4	13.6	46	74	26	-0.243	0.046
nad1	**EG**	29.9	10.2	14.9	45	74.9	25.1	-0.2	0.187
	**GW**	29.7	10.2	15	45	74.7	25.2	-0.21	0.190
	**HA**	29.7	10.2	15	45	74.7	25.2	-0.21	0.190
	**MA**	29.9	9.9	14.9	45.3	75.2	24.8	-0.21	0.2
	**NU1**	29.9	10.2	14.9	45	74.9	25.1	-0.2	0.187
	**NU3**	29.9	10.2	14.9	45	74.9	25.1	-0.2	0.187
	**SG**	29.9	10.2	14.9	45	74.9	25.1	-0.2	0.187
nad2	**EG**	32.9	6.8	11.4	49	81.9	18.2	-0.197	0.253
	**GW**	33.1	7	11.3	48.6	81.7	18.3	-0.19	0.235
	**HA**	33.1	7	11.3	48.6	81.7	18.3	-0.19	0.235
	**MA**	32.6	6.7	11.6	49.1	81.7	18.3	-0.2	0.268
	**NU1**	32.9	6.8	11.4	49	81.9	18.2	-0.197	0.253
	**NU3**	32.9	6.8	11.4	49	81.9	18.2	-0.197	0.253
	**SG**	32.9	6.8	11.4	49	81.9	18.2	-0.197	0.253
**nad3**	**EG**	33.2	9.3	9.3	48.1	81.3	18.6	-0.183	0
	**GW**	33.2	9	9.3	48.4	81.6	18.3	-0.186	0.016
	**HA**	33.2	9	9.3	48.4	81.6	18.3	-0.186	0.016
	**MA**	33.2	9.9	9.3	47.5	80.7	19.2	-0.177	-0.031
	**NU1**	33.2	9.3	9.3	48.1	81.3	18.6	-0.183	0
	**NU3**	33.2	9.3	9.3	48.1	81.3	18.6	-0.183	0
	**SG**	33.2	9.3	9.3	48.1	81.3	18.6	-0.183	0
**nad4**	**EG**	31.9	8.7	12.4	47	78.9	21.1	-0.191	0.175
	**GW**	32	8.7	12.3	47	79	21	-0.19	0.171
	**HA**	32	8.7	12.3	47	79	21	-0.19	0.171
	**MA**	31.8	9.1	12.5	46.6	78.4	21.6	-0.189	0.157
	**NU1**	31.9	8.7	12.4	47	78.9	21.1	-0.191	0.175
	**NU3**	31.9	8.7	12.4	47	78.9	21.1	-0.191	0.175
	**SG**	31.9	8.7	12.4	47	78.9	21.1	-0.191	0.175
**nad4l**	**EG**	31.4	7.5	11.8	49.3	80.7	19.3	-0.222	0.223
	**GW**	30.7	7.5	12.4	49.3	80	19.9	-0.233	0.246
	**HA**	30.7	7.5	12.1	49.7	80.4	19.6	-0.236	0.235
	**MA**	31	7.5	12.1	49.3	80.3	19.6	-0.228	0.235
	**NU1**	31.4	7.5	11.8	49.3	80.7	19.3	-0.222	0.223

	**NU3**	31.4	7.5	11.8	49.3	80.7	19.3	-0.222	0.223
	**SG**	31.4	7.5	11.8	49.3	80.7	19.3	-0.222	0.223
**nad5**	**EG**	32	9.3	12.5	46.2	78.2	21.8	-0.182	0.147
	**GW**	31.9	9.1	12.4	46.5	78.4	21.5	-0.186	0.153
	**HA**	31.9	9.1	12.4	46.5	78.4	21.5	-0.186	0.153
	**MA**	31.8	9.2	12.7	46.3	78.1	21.9	-0.186	0.16
	**NU1**	32	9.2	12.5	46.3	78.3	21.7	-0.183	0.152
	**NU3**	32	9.3	12.5	46.2	78.2	21.8	-0.182	0.147
	**SG**	32.1	9.4	12.4	46.1	78.2	21.8	-0.179	0.138
**nad6**	**EG**	29.2	6.2	13.1	51.5	80.7	19.3	-0.276	0.358
	**GW**	29.7	5.7	12.6	52	81.7	18.3	-0.273	0.377
	**HA**	29.7	5.7	12.6	52	81.7	18.3	-0.273	0.377
	**MA**	29.7	5.7	12.4	52.2	81.9	18.1	-0.275	0.37
	**NU1**	29.2	6.2	13.1	51.5	80.7	19.3	-0.276	0.358
	**NU3**	29.2	6.2	13.1	51.5	80.7	19.3	-0.276	0.358
	**SG**	29.2	6.2	13.1	51.5	80.7	19.3	-0.276	0.358
